# Dynamic Adaptive Cross-Chain Trading Mode for Multi-Microgrid Joint Operation

**DOI:** 10.3390/s20216096

**Published:** 2020-10-27

**Authors:** Longze Wang, Jing Wu, Rongfang Yuan, Delong Zhang, Jinxin Liu, Siyu Jiang, Yan Zhang, Meicheng Li

**Affiliations:** 1 State Key Laboratory of Alternate Electrical Power System with Renewable Energy Sources, School of New Energy, North China Electric Power University, Beijing 102206, China; 1182111018@ncepu.edu.cn (L.W.); zhangdelong@ncepu.edu.cn (D.Z.); 120192211823@ncepu.edu.cn (J.L.); 120202211047@ncepu.edu.cn (S.J.); mcli@ncepu.edu.cn (M.L.); 2 School of Economics and Management, North China Electric Power University, Beijing 102206, China; 120192206450@ncepu.edu.cn (J.W.); 120192206505@ncepu.edu.cn (R.Y.)

**Keywords:** cross-chain trading, consensus mechanism, key management interoperability protocol, multi-microgrid, blockchain

## Abstract

The emerging blockchain technology has injected new vitality into the energy market, especially the peer-to-peer power trading of microgrid systems. However, with the increase of energy blockchain projects, the difficulty of data communication and value islands between blockchain networks have become open issues. Thus, in this paper, we propose a dynamic adaptive cross-chain trading mode for multi-microgrid joint operation. The novelty is to design a proof of credit threshold consensus mechanism to achieve effective information verification. This consensus mechanism can ensure the adaptive consistency of cross-chain information without changing the existing blockchain architecture of each system. At the same time, we design a corresponding key management interoperability protocol based on RSA algorithm and Chinese remainder theorem, which can realize data transfer and information consensus for cross-chain transactions. The theoretical analysis verifies that the cross-chain communication information is effective and the system is able to protect against the attack of malicious nodes. Finally, a cross-chain simulation experiment is established to analyze the operation efficiency. The result shows that this cross-chain trading takes place within seconds, which basically meets the response requirements for multi-microgrid joint operation.

## 1. Introduction

The process of electrification has been greatly developed, and a complete microgrid system has been established in many areas [1]. Due to the utilization of renewable energy in microgrid systems, multi-microgrid joint operation often emerges in engineering for effective power scheduling of multi-entity systems [2]. Centralized operation always establishes a control center to gather the information of the whole system, and makes unified scheduling decisions [3]. However, exploiting a control center could be quite challenging for joint operation of multiple microgrids [4]. For example, a multi-microgrid system contains many stakeholders who do not want to be involuntarily regulated and worry about their privacy being disclosed [5]. Although distributed energy management is made for privacy-preserving [6], the joint operation could not realize trusted transactions of all stakeholders without authoritative intermediaries [7]. Therefore, it is difficult to set up a distributed network for the whole system, and to realize scheduling optimization of the multi-microgrid [8].

Blockchain technology has advantages of decentralized, trusted, transparency, fairness and can be applied to distributed decision-making [9], which is very suitable for establishing a trusted system of multiple stakeholders [10]. The blockchain-enhanced energy trading stands out to be a suitable solution [11,12]. For multi-microgrid joint operation, cross-chain technology of blockchain could break through the trust barrier of each microgrid and realize the energy transaction among multiple systems on the basis of protecting privacy [13]. Typical cross-chain technologies for asset transfer are based on four typical types: notary scheme, sidechains/relays, hash-locking and distributed private key control [14]. These methods mainly focus on asset mapping and asset transaction to reconstruct the value exchange network of blockchains [15]. However, it is difficult for public blockchain to provide more operable interface [16] and there is a single point of failure [17], so the existing cross-chain technology could not be applied to energy systems directly, especially unable to solve the cross-chain trading problem of multi-microgrid joint operation. Additionally, the main research gap are as follows:

1. The existing cross-chain technology mainly guarantees the atomicity of asset interaction by means of electronic cryptocurrency deployment. Due to the complex logic of upstream routing and asset interaction in general digital assets [15], it cannot be applied to the lightweight cross-chain dynamic adaptive interaction scenario of energy blockchains directly.
2. The underlying architecture and consensus mechanism of each energy blockchain network may be different [18]. Therefore, it is necessary to establish a universal and reliable dynamic adaptive cross-chain consensus mechanism without changing the original operation mechanism of each system.
3. The power system data is so huge and changes so instantaneously that it requires higher operation efficiency for multi-microgrid. The existing cross-chain key management pays little attention to data throughput and system operational efficiency.

According to the defect of existing technology, a dynamic adaptive cross-chain trading mode for multi-microgrid joint operation is proposed in this paper. This mode includes a novel cross-chain information verification consensus mechanism that can improve operational efficiency and an optimized key management interoperability protocol that can ensure privacy security. The main objectives and contributions of this paper are as follows:

1. We propose a proof of credit threshold consensus mechanism for cross-chain communication to achieve effective information verification. This consensus mechanism can ensure the adaptive consistency of cross-chain information without changing the existing blockchain architecture of each system.
2. We design a corresponding key management interoperability protocol though an optimized RSA algorithm based on Chinese remainder theorem, and this communication protocol can realize effective data transfer and information consensus for cross-chain transactions.
3. To analyze the feasibility of the cross-chain trading mode used in multiple energy systems, we verify the effectiveness, security and operational efficiency through theoretical and experimental results.

The organization of this paper is as follows. Section 2 reports some background and related work regarding this paper. Section 3 describes the cross-chain communication model for multi-microgrid joint operation. Section 4 presents the proof of credit threshold consensus mechanism, and Section 5 discusses key management interoperability protocol. Section 6 verifies the effectiveness and security of the cross-chain mode based on theoretical analysis. Section 7 analyses the operational efficiency through a case study. Finally, Section 8 summarizes the study and explores directions for future work.

## 2. Background and Related Work

In this section, we reviewed the state-of-the-art solutions for energy blockchain and cross-chain interoperability technology. We also presented the related work that supports the architecture proposed in this article.

### 2.1. Energy Blockchain

Blockchains are an emerging technology that has drawn considerable interest from startups, technology developers, financial institutions, national governments and the academic community [19]. As the underlying technology and infrastructure of Bitcoin, blockchain was proposed by Satoshi Nakamoto in 2008, the founder of Bitcoin [20]. Blockchain is a kind of distributed ledger that connects data blocks in chronological order and realizes secure data transmission and access by using cryptography technology [21]. In recent years, more and more scholars have begun to study the application of blockchain technology in the field of energy and developed many representative projects.

The first energy blockchain trading system was set up in Brooklyn, New York, which sells rooftop solar power from five households to another five households directly. This project had set a precedent of adopting blockchain technology in the energy sector [22]. Under the background of the industrial revolution, blockchain is employed to facilitate machine-to-machine (M2M) interactions and establish a M2M electricity market in the context of the chemical industry. The presented scenario includes two electricity producers and one electricity consumer trading with each other over a blockchain. This work contributes a proof-of-concept implementation of the scenario [23]. Since then, most of the energy blockchain research has focused on using the distributed data structure of blockchain to promote peer-to-peer energy transactions [12].

By using the blockchain technology to measure carbon emission rights, trading can be made more reliable. Literature [24] proposes a blockchain-based carbon emission rights verification system to learn proven data further by using the governance system analysis and blockchain mainnet engine to solve the problems in the process of carbon trading. In addition, with the use of blockchain technology in the field of electric vehicles, electric vehicle owners can gain enough transparency in energy management and have greater choice in selecting their energy supply [25].

Blockchain technology can clearly benefit energy system operations, markets and producers and sellers [11]. Energy blockchain is a rapidly developing research and development field. With the continuous deepening of energy blockchain research, the blockchain will inject new development vitality into the energy field.

### 2.2. Cross-Chain Interoperability

Due to the mutual independence of blockchains, it is difficult to carry out data communication and value transfer between blockchains, and value isolated islands gradually appears. Cross-chain interoperability technology is an important technical means for blockchain to realize interconnection and improve scalability [26]. Therefore, cross-chain interoperability technology has become a research hotspot, and four cross-chain interoperability technologies are proposed [14].

The notary scheme is the easiest way to interoperate between chains, which use a trusted entity as an intermediary between A and B [27]. Assuming that A and B can not trust each other, the entity that both A and B can trust is the intermediary of the notary. With the help of the intermediary, a top-level encryption hosting system is provided, which allows funds to flow between ledgers. The notary schemes can be divided into centralized notary schemes and multisig notary schemes [28].

The sidechains/relays method is a structure of one main blockchain plus one sub blockchain [15]. The sub blockchain can view some information on the main blockchain to complete the asset transfer and circulation on the two blockchains. In general, the main blockchain does not know the existence of the sub blockchain, but the sub blockchain must know the existence of the main blockchain.

The core technology of hash-locking is to provide the original contents of the unfrozen asset corresponding to the hash value within a specified period of time [14]. Hash locking realizes cross-chain transactions and information interaction by running specific smart contracts on two chains. As hash-locking schemes can be chained after each other, it is possible to enable trades even if there is no direct connection between the trading parties.

Distributed private key control can use private key generation and control technology to map encrypted currency assets to the chain based on the built-in asset template of the blockchain protocol, and deploy new smart contracts based on cross-chain transaction information to create new encrypted currency assets [29]. The user of the distributed private key and the decentralized network jointly control the private key, and there is no third party holding the private key.

Although the above cross-chain interoperability technologies have been applied in some fields, in general, blockchain cross-chain interoperability technology is still in its infancy [30]. In the future, more and more new technologies will be developed to deal with complex and diverse application scenarios.

### 2.3. Cross-Chain Technology in the Energy Field

Cross-chain technology would make a great contribution in the energy industry. However, as this research is just emerging, the related work is relatively few. This part lists all the existing cross-chain technology research in the energy field one by one.

Pop et al. [31] proposed a technique for tamper-evident registration of smart meters’ energy data and associated energy transactions using digital fingerprinting, which allows the energy transaction to be linked hashed-back on-chain, while the sensors data is stored off-chain. This study adopts a hash-locking method to improve the response time and data throughput for energy systems. In the proposed architecture, the authors innovatively introduce cross-chain interaction into power systems.

She et al. [32] proposed a model of multi-energy complementation and safety transaction on heterogeneous energy blockchain, which was based on a cross-chain mutual trust transaction method of relays mode. Such multiple energy transactions can be conducted directly between suppliers and consumers.

Ochôa et al. [33] described a cross-blockchain architecture that uses a protocol widely used in the smart grid scenario, supporting the implementation of this architecture on existing systems. The presented architecture provides security, reliability and privacy for users through the use of sidechain communication technology.

Firoozjaei et al. [34] proposed a hybrid blockchain with subnetwork that aims to protect the privacy of users and provides a trusted workflow for billing and charging transactions. This work introduced the credit-sharing feature for energy markets in the service layer, and the subnetwork blockchain information of users’ peer-to-peer energy transactions in the microgrid is anonymized by the bridge and no private information is leaked to the main power grid. 

He et al. [35] described a joint operation system of cross-chain trading, combined distributed photovoltaic power generation market and the carbon market. To share data and circulate value for two markets, the authors designed a two-way anchoring method that achieves equating between carbon trading and electricity trading by cryptocurrency. The authors used a structure of the main chain plus a side chain to achieve cross-chain communication.

Table 1 shows a comparison synthesis of the related works. In Table 1, we list the applications of all current blockchain cross-chain interoperability technologies in the energy field, and compare their technical types, technical contribution and application scenarios.

None of the related work presented shows in detail the consensus proof of cross-chain communication and how this can change the interoperability protocol of blockchain architecture. Our work used threshold scheme and credit evaluation to build a consensus mechanism suitable for dynamic adaptive cross-chain trading. A key management interoperability protocol was designed to realize privacy protection and data traceability for multiple stakeholders. In addition, we verified the performance of the proposed cross-chain trading mode such as effectiveness, security and operational efficiency.

## 3. Cross-Chain Transaction for Multi-Microgrid

**Figure 1:**
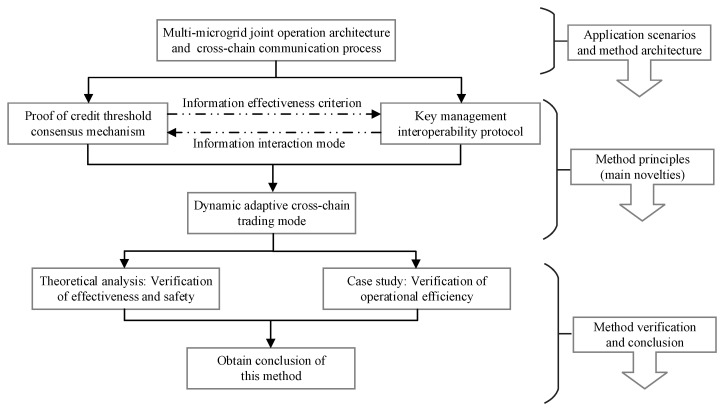
The idea organization and main procedures.

Up to now, there are many projects on the microgrid internal trading based on blockchain, but in fact, the research of multi-microgrid joint operation using blockchain can not be ignored. The main objective of this study is to design a dynamic adaptive cross-chain trading mode to realize the multi-microgrid joint operation. Figure 1 shows the idea organization and main procedures of this study.

The proposed method takes multi-microgrid joint operation as the application scenarios, and the basic architecture of our method was first introduced. Secondly, we proposed a novel proof of the credit threshold consensus mechanism and the corresponding key management interoperability protocol to construct the dynamic adaptive cross-chain trading mode. Additionally, the consensus mechanism and the interoperability protocol are the main novelties of our method. Finally, we verified the feasibility of the proposed method and obtained the conclusion of this work.

### 3.1. Multi-Microgrid Joint Operation Architecture

**Figure 2:**
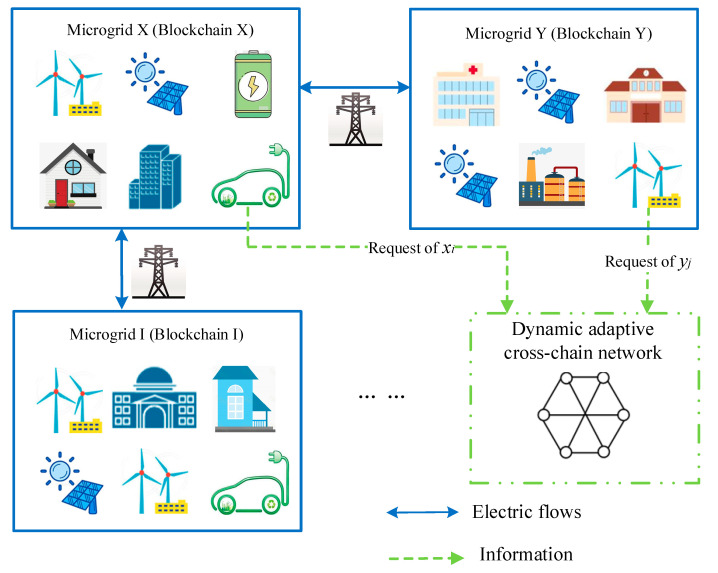
Multi-microgrid joint operation architecture.

The microgrid is a small power distribution system composed of distributed power supply, storage device, electrical load, monitoring and protection device. This concept provides a new solution for flexible and efficient utilization of distributed renewable sources [36]. With the large-scale construction of microgrid, multiple adjacent microgrids in a certain region can form a multi-microgrid joint operation system through the connected transmission and distribution network [37]. Figure 2 illustrates the trading architecture of the multi-microgrid joint operation.

Figure 2 shows two independent microgrid systems X and Y, and the transmission and distribution network in this region constructed a physical channel for power interaction. The two microgrids had respectively built internal peer-to-peer power trading networks based on blockchain, namely blockchain X and blockchain Y. In general, each individual microgrid buys power at a higher price or sells power at a lower price to an external distribution network when the electric power is unbalanced. This approach is not conducive to the economic operation of each microgrid, in that long-distance transmission in the distribution network leads to energy losses. Therefore, we designed a dynamic adaptive cross-chain network, so that each stakeholder in blockchain X and Y can conduct independent cross-system transaction. Since this dynamic adaptive network is established by blockchain technology, cross-chain trading shares the advantages of blockchain such as distributed data storage, transparent information, decentralized mutual trust and so on.

Since each energy blockchain is independent, data communication and value transfer between multi-microgrids are still facing challenges. Therefore, we proposed a dynamic adaptive cross-chain communication process, which could realize multi-microgrid joint operation.

### 3.2. Cross-Chain Communication Process

**Figure 3:**
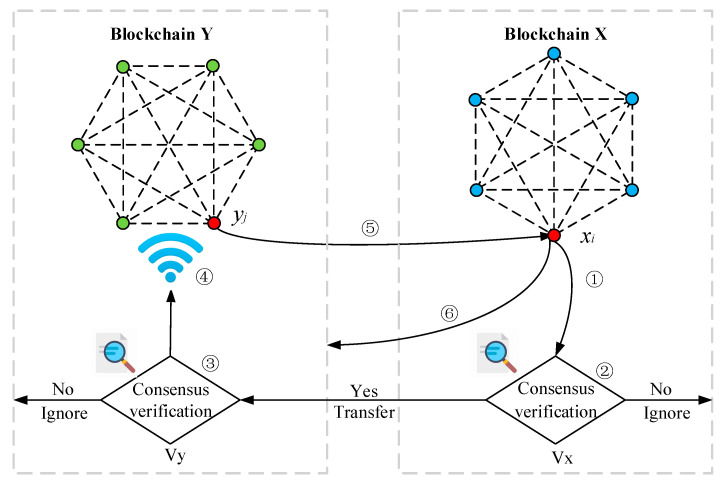
Cross-chain communication process for multi-microgrid.

The cross-chain communication process of blockchain X and Y is shown in Figure 3. There are multiple nodes of power production, sales and consumption in both energy blockchain networks, in which:

(1) $$\{\begin{array}{c} X\in \left(x1,x2,\cdots ,xn,x\in X\right),n>0\\ Y\in \left(y1,y2,\cdots ,ym,y\in Y\right),\;m>0 \end{array}$$

In each blockchain network, a verification nodes subgroup (VNS) was selected to verify the validity of cross-chain communication information, such as {Vx} and {Vy}. VNS was selected according to credibility, and the specific method will be described in Section 4. {Vk} is the cross-chain consensus VNS for multi-microgrid joint operation, and it is constituted by {Vx} and {Vy}:

(2) {Vk}∈Vx∪Vy

A node $x_{i}$ in microgrid X sends cross-chain trading requirements, and a node $y_{j}$ in microgrid Y accepts and enters into a trading agreement. This process consists of six stages:

1. $x_{i}$ constructs a figure identity certificate and a data transfer key $x_{i,s}$, and then writes the description of the cross-chain trading requirements $mx_{i}$. The details of the power transaction and the deadline are written into a smart contract $ET_{x_{i}}^{request}$, and this smart contract is deployed.
2. {Vx} verifies the trading requirement $ET_{x_{i}}^{request}$ by the consensus mechanism presented in Section 4. If the verification is successful, the key is updated and a new smart contract is built. Then the cross-chain transaction requirements are sent to {Vy}. If the verification is not passed, the trading requirement $ET_{x_{i}}^{request}$ is ignored.
3. {Vy} verifies the trading requirement $ET_{x_{i}}^{request}$. If the verification is successful, the key is updated and a new smart contract is built. The smart contract broadcasts to all nodes in blockchain Y. If the verification is not passed, $ET_{x_{i}}^{request}$ is ignored.
4. The node $y_{j}$ in blockchain Y is expected to conclude a deal with $x_{i}$ based on the trading requirement $ET_{x_{i}}^{request}$. If Equations (3)–(5) are true, $y_{j}$ updates the key and writes the blind response result Reply($mx_{i}$). Then deploy the corresponding power transaction $ET_{x_{i},\;y_{j}}^{response}$ replay to $x_{i}$. If Equations (3)–(5) are false, the power transaction is ignored.
  (3) $$t_{current}\leq t_{contract_{deadline}}-\Delta t$$
  (4) $$\mathrm{Request}\;\left(mx_{i}\right)\in \left\{Vy\right\}$$
  (5) $$\mathrm{Traceability}\;\left(mx_{i},\;Key,\;sig\right)==1$$
  where Δt indicates the upper limit of the time to deploy or execute a smart contract, or indicates the upper limit of single-hop response time. $\mathrm{Traceability}\;\left(mx_{i},Key,\;sig\right)$ is the proof of information traceability and trustworthiness. Key and sig respectively indicate the key and corresponding digital signature of $x_{i}$ to $y_{j}$.
5. $x_{i}$ verifies the smart contract $ET_{x_{i},\;y_{j}}^{response}$ from $y_{j}$. If it passes, $x_{i}$ writes the hash function of power trading information into the smart contract and extracts the response result Reply($mx_{i}$). $x_{i}$ returns the power transaction key to $y_{j}$ when the smart contract executes. Other cross-chain transactions are executed in turn similarly to the above stages.
6. After the power transaction protocol is executed, $x_{i}$ and $y_{j}$ broadcast the cross-chain certificate to the multi-microgrid system respectively.

In the above-mentioned cross-chain communication process, it is necessary to establish a cross-chain consensus mechanism to achieve decentralized, dynamic and adaptive transfer of values between the nodes of different blockchains. In addition, in order to ensure the security and trustworthiness of data transmission, it is necessary to design a key management interoperability protocol for cross-chain communication.

## 4. Proof of Credit Threshold Consensus Mechanism

The consensus mechanism is a mechanism to ensure the consistency of honest node data in a decentralized system. Its purpose is to create a reliable and tamper-proof decentralized network and to ensure the data safety [38]. Currently, common consensus mechanisms of blockchain mainly include proof of work (PoW), which first appeared in the Bitcoin system designed by Satoshi Nakamoto, and proof of stake (PoS), which first appeared in the PPcoin. The POW consensus mechanism works very slowly, processing only seven transactions a second [39]. At the same time, the nodes will consume a large amount of computing power, that is, a large amount of electric energy. This is inconsistent with the original intention of microgrid. In the POS consensus mechanism, the probability of the node gaining the accounting right will increase with the increase of the node’s stake. Since there are relatively few nodes in the microgrid, nodes with high stake may make the blockchain bipartite or double payment. Nodes with a low stake may be unable to participate in the consensus process, which affects the security of the energy blockchain [40]. Therefore, the existing consensus mechanism based on POW and POS cannot adapt to the power transaction of microgrid, especially the cross-chain trading mode for multi-microgrid joint operation.

Some privileged nodes with high credibility can be selected to verify cross-chain information, which can guarantee the efficiency and trustworthiness of cross-chain consensus. Moreover, the cross-chain communication from a single node is stored in multiple verification nodes through distributed key and threshold digital signature, which is conducive to secure consensus verification. Therefore, in the cross-chain power trading scenario, we proposed the proof of credit threshold (PoCT) consensus mechanism based on threshold signature scheme and credit evaluation.

### 4.1. Threshold Signature for Cross-Blockchain Communication 

It is necessary to authorize a part of nodes to form the {Vk} in the cross-chain communication for multi-microgrid joint operation. The full node server in the system is responsible for maintaining {Vk} and providing them with all valid operational information until a protocol is reached. A new data block generation is completed when the nodes of {Vk} reach a consensus result. 

The number of nodes in {Vk} can be selected according to the actual situation of multi-microgrid system. Based on the results of credit evaluation, multiple VNSs are selected from each blockchain network, such as {Vx} and {Vy}. These VNSs extend the intra-chain consensus of each blockchain into inter-chain consensus through threshold signature. The nodes in VNS are independent individuals, mutually disjoint and influence. The public-private key pair ($K_{V}^{public},\;K_{V}^{private}$) of {Vk} is generated according to threshold signature scheme, and the cross-chain consensus rules are as follows:

(6) $$\left\{Vk\right\}=\left\{V_{1}||V_{2}||\cdots ||V_{k},\;V_{i}||V_{j}=\emptyset \right\},\;\left(1\leq i,\;j\leq k\right)$$

(7) $$\left|Vk\right|=n_{k}\;(n_{k}\geq 0)$$

(8) $$\sum_{k=1}^{m}n_{k}=n\;\left(m\geq 1\right)$$

(9) $$CK_{V}^{public}\left(ET,\;t_{k},\;n_{k}\right)=\{\begin{array}{cc} \mathrm{True}, & \mathrm{if}\;n_{k}\geq t_{k}^{\prime }\geq t_{k}\\ \mathrm{False}, & \mathrm{otherwise} \end{array}$$

(10) $$CK_{V}^{public}\left(ET,t_{1},n_{1};\cdots ;t_{m},n_{m};t,n\right)=\{\begin{array}{cc} \mathrm{True}, & \mathrm{if}\;n_{i}\geq t_{i}^{\prime }\geq t_{i}\;\sum_{i=1}^{m}t_{i}\geq t\\ \mathrm{False}, & \mathrm{otherwise} \end{array}$$

where $n_{k}$ indicates the number of all nodes in {Vk}, and $t_{k}$ indicates the minimum number of $n_{k}$ required to pass a certain verification. $t_{k}^{\prime }$ represents the actual number of $n_{k}$ that pass a certain verification. These rules conform to the (*t*, *n*) threshold multi-signature scheme, which is to say, *n* nodes in {Vk} can verify information and at least *t* nodes are required to pass a certain verification. In the above-mentioned cross-chain consensus rules, Equation (6) defines the cooperation relationship between each independent blockchain, and Equations (7) and (8) describe the scale of cross-chain consensus verification nodes. Equation (7) represents the threshold condition for an energy blockchain to verify the cross-chain information. Accordingly, Equation (8) verifies the cross-chain consensus condition by the threshold signature.

The threshold signature scheme is a name for the combination of distributed key generation (DKG) and threshold multi-signature. It has significant advantages such as low cost, high security, data credibility and expansion space, and is widely used in smart contracts of blockchain. Threshold signature scheme is introduced in multi-microgrid cross-chain communication to carry out distributed key management, which can improve the security of power data transmission. Moreover, a few privileged nodes reach a consensus to verify the cross-chain communication, which can improve the operation efficiency in the multi-microgrid system. We selected a privileged verification nodes group through the credit evaluation conforming to the microgrid power transaction.

### 4.2. Credit Evaluation of Microgrid Power Transactions 

An attribute set $Set_{i}$ is introduced for all nodes in order to express the credit evaluation of each stakeholder in the multi-microgrid system, and it is expressed as follows:

(11) $$Set_{i}=\langle a_{i},\;b_{i},\;c_{i}\rangle$$

where $a_{i}$ indicates the address information of node *i*, and $b_{i}$ indicates the account balance and $c_{i}$ is to evaluate a node credit in terms of cross-chain transactions.

The default of cross-chain trading protocol generally has two categories when the smart contract of cross-chain trading is signed. If the actual amount of electricity provided by the supplier in this time slot is lower than the amount stipulated in the contract, users would buy the lack electricity from the grid. Since grid prices are generally higher than contract prices, these users suffer economic losses. On the other hand, if users actually use less than the amount of electricity in contracts, the surplus electricity used would be bought by the grid. As a result, the grid purchase price was lower than the contract price, which caused economic loss to suppliers. Therefore, it needs to consider the contract performance by suppliers and users to evaluate the credit. The credit evaluation of supplier *i* and user *j* was measured as follows:

(12) $$C_{i}^{supplier}=\{\begin{array}{cc} C_{i-1}^{supplier}-1, & Q_{i,j}<Q_{i,j}^{actual}\\ C_{i-1}^{supplier}, & Q_{i,j}>Q_{i,j}^{actual} \end{array}$$

(13) $$C_{j}^{user}=\{\begin{array}{cc} C_{j-1}^{user}-1, & Q_{i,j}>Q_{i,j}^{actual}\\ C_{j-1}^{user}, & Q_{i,j}<Q_{i,j}^{actual} \end{array}$$

where $C_{i}^{supplier}$ and $C_{i-1}^{supplier}$ indicate the credit evaluate of supplier *i* in this and the last cross-chain transaction, and $C_{j}^{user}$ and $C_{j-1}^{user}$ indicate the credit evaluate of user *j* in this and the last cross-chain transaction. $Q_{i,j}$ indicates the amount of electricity required by the smart contract, and $Q_{i,j}^{actual}$ indicates the actual amount of electricity. Contract credit evaluation of suppliers and users are collectively expressed as $C_{i}^{contract}$, which denotes the executive condition of trading protocol at a time gap.

In the process of participating in the consensus, nodes take the digital signature into a block, which is the process of verifying the block and voting. There are two kinds of digital signatures including virtuous votes and vicious votes. The virtuous vote is nodes to verify the block to meet the system difficulty, and make legal signature. The vicious vote is a signature that does not conform to regulations, and the last node packs the data with signature into the block and broadcasts it in the blockchain network regardless of the illegal behavior. Once the other nodes verify that the signature is not valid, it is stipulated that all nodes signed on the block have a vicious vote. Vicious voting behavior needs to be considered when electing privileged nodes for cross-chain consensus. The credit evaluation of a node voting behavior is measured as follows:

(14) $$C_{i}^{vote}=\frac{1}{1+e^{\gamma \sum_{k=0}^{N}\alpha_{k}-\sum_{k=0}^{N}\beta_{k}}}$$

where $C_{i}^{vote}$ is the vote credit evaluation of node *i* in the time gap *m*. *N* indicates total number of time gaps experienced. $\alpha_{k}$ and $\beta_{k}$ respectively indicate the vicious vote and the virtuous vote within *k* time gaps. γ is a penalty factor. If it has a larger number, the less tolerant of the system would be to vicious voting behavior. In general, the number of γ is between 1 and 5.

According to the $C_{i}^{contract}$ and $C_{i}^{vote}$, the cross-chain credit evaluation $c_{i}$ can be measured as follows:

(15) $$C_{i}^{m,original}=C_{i}^{contract}C_{i}^{vote}$$

(16) $$C_{i}^{m^{\prime }}=\lambda \;C_{i}^{m,original}+\left(1-\lambda \right)C_{i}^{m-1^{\prime }}$$

(17) $$c_{i}^{m}=C_{i}^{m^{\prime }}e^{-\partial l}$$

where $C_{i}^{m,original}$ is the product of $C_{i}^{contract}$ and $C_{i}^{vote}$, which does not correctly assess the credit evaluation because $C_{i}^{m,original}$ grows too fast with the number of growth for cross-chain transactions. Therefore, considering that the transaction of this time gap is affected by the previous time gap, the node attribute after time correction $C_{i}^{m^{\prime }}$ can be calculated. λ is a correction coefficient and its initial value is 1, which is used to avoid the node credit attribute rising too fast in the trading period.

If fewer nodes participate in cross-chain trading, consensus efficiency and information verification would be affected. In order to ensure the activity of each node in cross-chain trading, activity loss is introduced in the credit evaluation. As long as the node participates in cross-chain transaction in a time gap, the $c_{i}$ of this node would not produce direct loss. The calculation method of $c_{i}^{m}$ is shown as Equation (17) after increasing the activity loss. ∂ indicates a penalty factor for not actively participating in cross-chain trading, and l indicates the number of time gaps in continuous non-participation in cross-chain trading.

To sum up, the credit values $c_{i}$ of each node in each time gap can be calculated by comprehensively considering the factors such as implementing smart contracts, voting behavior and cross-chain trading initiative. The multi-microgrid system sorts the $c_{i}$ of each node, and selects a number of top ranked nodes according to the threshold signature scheme as privileged nodes to verify cross-chain communication. This mode constitutes the PoCT consensus mechanism of cross-chain communication in multi-microgrid joint operation. In order to realize the consensus mechanism in cross-chain communication, a corresponding key management interoperability protocol, which is suitable for energy blockchain is required.

## 5. Key Management Interoperability Protocol in Cross-Chain

Key management interoperability protocol (KMIP) is a kind of communication protocols, which defines how to manipulate encrypted key messages on the secret key management server and network. KMIP supports symmetric and asymmetric encryption, and performing encryption operations on distributed networks, such as encryption and decryption [41]. Constructing a secure and efficient KMIP can realize the effective transmission and consensus of cross-chain trading information. An optimized RSA algorithm adopted Chinese remainder theorem (CRT) is proposed, which can improve the decryption operation speed to adapt to the real-time response of power data transmission. Based on the optimized RSA algorithm, a cross-chain communication KMIP is built to guarantee privacy protection and data traceability. 

### 5.1. Optimized RSA Algorithm based on Chinese Remainder Theorem

RSA algorithm is a cryptosystem constructed based on number theory proposed by R. Rivest, A. Shamir and L. Adleman in 1978, and it is the most mature and perfect public key cryptosystem so far [42]. The principle of this algorithm is finding two large prime numbers easily, but it is extremely difficult to factor their product. Therefore, the product can be exposed as an encryption key. RSA algorithm can be divided into three processes: key generation, encryption and decryption, and CRT is used to optimize the decryption process of this algorithm.

In the key generation process, two secret large prime numbers *p* and *q* are selected, then *r* and φ(r) are calculated by Equations (18) and (19). φ(r) is the Euler’s function of *r*. Select an integer *e* so that *e* satisfies the Equation (20). Finally, *d* is calculated by Equation (21), and it is the multiplicative inverse based on φ(r). In that *e* and φ(r) are relatively prime, there must be a multiplicative inverse of *e* according to modular arithmetic. Therefore, {e,r} indicates public key and {d,r} indicates private key. Equations (18)–(21) are shown as follows:

(18) r=p×q

(19) φ(r)=(p−1)(q−1)

(20) gcd(φ(r), e)=1, 1<e<φ(r)

(21) d·e≡1 mod φ(r)

In the encryption process, RSA algorithm is a typical asymmetric encryption algorithm, in which the one-way function forward solution is very simple, but the reverse solution is very complex. The plaintext bit string is first grouped in the encryption process. This makes each grouping corresponding decimal number less than *r*, and the block length is less than $\log_{2}r$. Then plaintext m is grouped and encryption is measured as follows:

(22) $$c\equiv m^{e}\;mod\;r$$

where *c* is the ciphertext produced by the public key to encrypt the information *m*. 

The decryption operation of traditional RSA algorithm for ciphertext packet is as follows:

(23) $$c\equiv m^{d}\;mod\;r$$

This decryption process requires the calculation of an integer to an integer power, and then takes its modulus. The intermediate result of the direct calculation is very large and may exceed the range of integer values allowed by computers. Even if it can be solved by an intelligent computer, the long calculation time cannot meet the instantaneous response requirement for electricity trading.

Chinese remainder theorem (CRT) is an important theorem in number theory, which was used in ancient China to solve a group of congruences. CRT can be summarized in two rules. Rule 1: Adding two numbers. If there is an addend that is not divisible by an integer A, then their sum is not divisible by the integer A. Rule 2: Two numbers are not divisible. If the divisor expands (or shrinks) several times while the dividend remains the same, the quotient and remainder increase (or shrink) by the same multiple, and the remainder must be less than the divisor [43]. 

The decryption party’s verification calculation is as follows:

(24) $$d_{p}\equiv d\;mod\;\left(p-1\right),\;d_{q}\equiv d\;mod\;\left(q-1\right)$$

(25) $$m_{p}\equiv c_{p}^{d}\;mod\;p,\;m_{q}\equiv c_{q}^{d}\;mod\;p$$

Based on CRT, *m* can be measured as follows:

(26) $$\{\begin{array}{c} m_{p}\equiv c_{p}^{d}\;mod\;p\equiv c^{d}\;mod\;p\equiv m\;mod\;p\\ m_{q}\equiv c_{q}^{d}\;mod\;q\equiv c^{d}\;mod\;q\equiv m\;mod\;q \end{array}$$

After CRT optimization, the whole decryption time is focused on solving $m_{p}$ and $m_{q}$ instead of solving *r* modulus. $m_{p}$ and $m_{q}$ are calculated by the typical binary representation algorithm and recursive remainder sum algorithm. The remainder table changes from a table of size 2*r* × *r* to two tables of modulus for *p* and *q* that has a calculated amount of $2\times \left(\frac{1}{2}n\right)\left(\frac{1}{2}n\right)$. The decryption process takes only half as much computation. Therefore, if the CRT calculation cost is not considered, the decryption operation speed of the optimized method is 4 times that of the original decryption operation speed. If the CRT calculation cost is considered, the decryption operation speed is 3.32 times that of the conventional RSA algorithm [44]. The time of decryption process is significantly reduced, which can improve the operation efficiency of cross-chain trading.

### 5.2. Key Management Interoperability Protocol 

It is necessary to allocate the public key of cross-chain information reasonably for multi-microgrid joint operation. Generally, users publish their public keys by sending to others, or broadcasting to a group. For example, in the Internet mailing list, a user attaches his or her public key to a message and sends it to a public area. While this method is simple, it has a major drawback that anyone can fake such publicly published information.

In view of the above-mentioned defection and combined with the PoCT consensus mechanism, we took the set of cross-chain consensus verification nodes {Vk} as the management organization of cross-chain public keys. This organization is equivalent to a public key dynamic directory table, and has the functions of establishing, maintaining and publishing public keys. Since {Vk} is dynamically elected based on the credit evaluation, the members in this set would be more reliable so that the security of key management can be higher.

**Figure 4:**
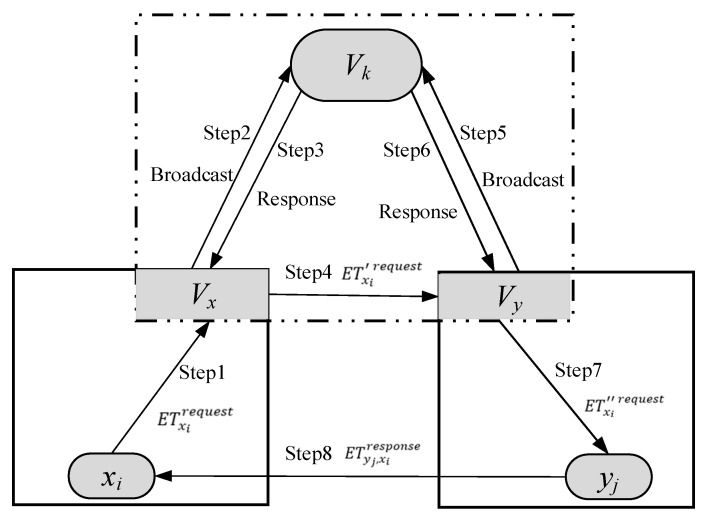
The process of key management interoperability protocol (KMIP) in the cross-chain trading.

Figure 4 shows the process of a node in the blockchain X sending a transaction requirement to blockchain Y and finally reaching a trading protocol. The process of KMIP can be divided into eight steps in the cross-chain trading. 

Step 1. A node $x_{i}$ in the blockchain X sends a cross-chain trading requirement to blockchain Y. Firstly, $x_{i}$ encapsulates the request data and sends it to {Vx}, which is the VNS of blockchain X. Then a smart contract $ET_{x_{i}}^{request}$ is generated and deployed in the {Vx}. The key transfer for this smart contract is as follows:

(27) $$x_{i}\overset{ET_{x_{i}}^{request}}{\to }\left\{Vx\right\}:ET_{x_{i}}^{request}=mx_{i}∥m^{e}\left(mx_{i}\right)$$

Step 2. The verified information from {Vx} is broadcast to the whole cross-chain consensus nodes group {Vk}. This information contains a request to obtain the current public key of blockchain Y. It is worth noting that {Vx} and {Vy} are all the subset of {Vk}.

Step 3. The response of {Vk} to $ET_{x_{i}}^{request}$ can be presented by a smart contract, which uses a private key $K_{V}$ to encrypt. So, {Vx} can decrypt using the public key of {Vk} and enable {Vx} to trace the information. There are three aspects of information in the response smart contract. The first is $K_{y}^{public}$ that is the public key of {Vy}, and it can be used by {Vx} to encrypt request information. The second is $ET_{x_{i}}^{request}$ that is trading the request sent from {Vx}, and it can be used to verify the received reply is indeed the response of this request. The last one is the timestamp that makes {Vx} believe the broadcast contract is not an old message. Therefore, the public key in the smart contract is indeed the current public key of {Vy}.

Step 4. {Vx} encrypts $ET_{x_{i}}^{request}$ with the public key $K_{y}^{public}$, and sends it to {Vy}. The sent smart contract consists mainly of two messages, including specific transaction request $ET_{x_{i}}^{request}$ and a random private key that is used to indicate the uniqueness. The update smart contract is as follows:

(28) $$x_{i}\overset{ET_{x_{i}}^{\prime }{}^{request}}{\to }\left\{Vx\right\}:ET_{x_{i}}^{\prime }{}^{request}=mx_{i}∥m^{d}\left(ET_{x_{i}}^{request},\;t_{x},\;n_{x}\right)$$

where $n_{x}$ indicates the total number of {Vx} and $t_{x}$ indicates the minimum number of verification nodes in {Vx}.

Step 5 and 6. {Vy} in blockchain Y obtains the public key $K_{x}^{public}$ of {Vx} in the same key management mode as Steps 2 and 3. This time, {Vx} and {Vy} have obtained each other’s public key and can communicate securely in the cross-chain network.

Step 7. {Vy} broadcasts the updated message to blockchain Y and is measured as follows:

(29) $$\left\{Vy\right\}\overset{ET_{x_{i}}^{\prime \prime }{}^{request}}{\to }y_{j}:ET_{x_{i}}^{\prime \prime }{}^{request}=mx_{i}∥m^{d}\left(ET_{x_{i}}^{\prime }{}^{request},\;t_{x},\;n_{x};t_{y},\;n_{y};t,\;n\right)$$

Suppose $y_{j}$ firstly responds to the trading request and validates that the trade request passed. For that {Vy} and {Vk} have verified $ET_{x_{i}}^{request}$, the corresponding path-proof is stored and updated in the private key of the trading request, including $t_{y},\;n_{y},t,\;n$.

Step 8. A response node $y_{j}$ verifies $ET_{x_{i}}^{\prime \prime }{}^{request}$. After the verification, an update smart contract $ET_{y_{j},\;x_{i}}^{response}$ is constructed according to the characteristics of power trading and is send to $x_{i}$. All the information during the key management interoperability is also written into the smart contract as follows:

(30) $$y_{j}\overset{ET_{y_{j},\;x_{i}}^{response}}{\to }x_{i}:ET_{y_{j},\;x_{i}}^{response}=my_{j}∥m^{d}\left(ET_{x_{i}}^{\prime \prime }{}^{request}\right)$$

where $x_{i}$ decrypts the smart contract according to Equation (26). If the decrypted information matches the trading request sent, a cross-chain protocol can be reached with $y_{j}$. 

## 6. Theoretical Analysis

The effectiveness, security and operational efficiency are three important evaluation indexes in the cross-chain trading mode. This section analyzed the indexes of effectiveness and security through detailed theoretical analysis.

### 6.1. Effectiveness Analysis

The effectiveness of cross-chain communication can be analyzed from three aspects, including encryption/decryption, key interoperability and consensus signature.

The effectiveness of encryption/decryption. The public key for cross-chain communication is {e,r} in the multi-microgrid joint operation, where *e* is a prime number randomly specified by the system, and *r* is calculated after a user has all the factorizations of the public key. Each cross-chain consensus verification node can only host the prime factorization factor of *r*. The number of prime factorization factors for *r* is the same as the number of cross-chain consensus verification nodes. Moreover, gcd(φ(r), e)=1 is based on Equation (20). Therefore, in the case that the factorization of e and *r* is determined, we could measure whether the arithmetic solution equals 1, namely [e,φ(r)]=1, according to the first congruence equation theorem. Then it can be calculated whether a unique user private key {d,r} exists. For plaintext information m, Euler’s theorem is applied to calculate as follows:

(31) $$D\left[E\left(m\right)\right]=D\left(c\right)=c^{d}\;mod\;r\equiv m^{e,d}\;mod\;r\equiv m^{k\varphi \left(r\right)+1}\;mod\;r\equiv m\;mod\;r$$

The same argument can be made E[D(m)]=m. Therefore, E[D(m)]=D[E(m)]=m. It can be shown that the process used for encryption/decryption follows the basic RSA principle in the cross-chain communication. Therefore, the encryption and decryption are effective.

The effectiveness of key interoperability. Suppose that *A* and *B* are attached to different microgrid nodes and want to conduct cross-chain communication. According to KMIP proposed, both *A* and *B* know each other’s public information parameters $parm_{A}$ and $parm_{B}$, and each has its own private key. Based on the basic principle of blockchain hash function, *A* and *B* can obtain communication key as follows:

(32) $$Key_{A}=Hash\left(K_{A}^{private}\cdot parm_{B}\right)$$

(33) $$Key_{B}=Hash\left(K_{B}^{private}\cdot parm_{A}\right)$$

Due to multiplication commutativity, we could measure that $Key_{A}=Key_{B}$. Therefore, the key interoperability is effective.

The effectiveness of the consensus signature is mainly analyzed from the power trading request to the signature of cross-chain consensus verification. According to the above-mentioned PoCT consensus mechanism, the nodes participating in the cross-chain verification all elect the members with high credibility through dynamic voting. Among these privileged nodes, only when more than or equal to *t* nodes verify the message successfully, can a consensus of cross-chain communication be reached. At the same time, the behavior of these nodes is stored in the digital signature, and the credit evaluation would be lower if there is vicious activity. If the credit evaluation is lower than the threshold value, the node would be excluded from the privileged node set. This way ensures the trustworthiness of the cross-chain consensus verification. Therefore, the cross-chain consensus signature is effective.

### 6.2. Security Analysis

The security of cross-chain joint operation can be analyzed from three aspects, including key confidentiality, forward/backward confidentiality and node hazard resistance.

Key confidentiality means that an attacker cannot learn any key information in an open source environment. The newly issued trading requests from nodes are non-interactive in key sharing of cross-chain joint operation. This means that there is no leakage of key information during the transmission of the trading request. In addition, the proposed KMIP is based on the RSA algorithm optimized by CRT. It is extremely difficult to crack the key without information, that is, there is no algorithm to find the private key in the polynomial. Therefore, the key confidentiality is security.

For forward/backward confidentiality, attention must be paid to prevent malicious nodes from obtaining new keys through old keys. In the proposed cross-chain mode, the key management of nodes is independent of each other, and the interoperability protocol built by a new trading request is independent. Even if an attacker knows one key or a subset of the keys assigned to it, the attacker cannot obtain another key. Therefore, the proposed KMIP provides confidentiality both forward and backward. Moreover, the PoCT consensus mechanism only selects nodes with higher credit evaluation, so that these trusted privileged nodes have a chance to obtain more cross-chain data, and nodes with malicious behavior will be excluded from the threshold strategy.

Node hazard resistance is the ability to resist or tolerate attacks in the cross-chain interoperability protocol. The node hazard resistance of the proposed mode can be proved as follows:

(34) $$d\equiv e^{-1}mod\;\varphi \left(r\right)\equiv e^{-1}mod\prod_{i\in t}group\;\varphi_{t}$$

(35) $$m=m^{d}\left(ET_{i},sig_{1},sig_{2},\cdots ,sig_{t}\right)$$

where *sig* indicates digital signature of each cross-chain consensus nodes for the trading message. According to the above equations, if a node wants to obtain the key *d*, it must obtain *t* subkeys from the distributed network. Therefore, this mode is completely secure unless an external malicious node simultaneously breaks *t* or more nodes. This scheme can resist the *t* − 1 nodes being broken.

## 7. Case Study

### 7.1. Experimental Deployment

To test the operational efficiency of cross-chain transaction based on PoCT and KMIP mechanism proposed in this paper, we chose the energy blockchain project in Brooklyn, New York as the experimental scenario. This project is the first peer-to-peer power transaction project based on the blockchain in the world, and has grown from an initial 10 distributed photovoltaic power suppliers to about 60 power supply and consumption units. Taking this real case as the experimental scenario, we constructed two blockchain-based microgrid systems, in which each microgrid contained 60 energy units. The two microgrids can interact with electricity across systems, and each energy blockchain has the same composition as the Brooklyn project. 

We assumed the two energy blockchains X and Y based on the above physical basis. *Nx* and *Ny* respectively indicates the total number of nodes inside blockchain X and Y. *Vx* and *Vy* respectively indicate the number of nodes for cross-chain consensus verification inside blockchain X and Y, and *Vk* is the total set of verify nodes that is *Vk* = *Vx* + *Vy*. Moreover, the number of transactions in every consensus is expressed as *τ*, and the system constructs cross-chain transactions at intervals of 1000 ms.

The simulation experiment is deployed and run on the Ethereum, which was established in 2014 by Vitalik Buterin through a crowdfunding project. Ethereum is an open source blockchain network and is able to execute smart contracts and to store them. Smart contracts executed on the Ethereum have flexible compilation, high efficiency, low cost and other advantages. We built an Ethereum simulation test environment consisting of 120 virtual nodes and 60 verify nodes on a computer. The computer configuration is as follows: CPU, Intel Core i7; memory size, 16 GB; operating system, Windows 10.

The underlying architecture for the multi-microgrid joint operation is first coded, including the above-mentioned consensus mechanism and key management protocol. The smart contracts for basic operations were then uploaded, installed and deployed on this cross-chain architecture to be further invoked. We deployed experiments according to the actual operation of the Brooklyn project, including power transaction demand release, transaction agreement reached, P2P power dispatch and settlement. On the other hand, we adjusted the trading parameters to achieve different operating efficiencies and to discuss the results.

### 7.2. Experimental Results and Discussions

**Figure 5:**
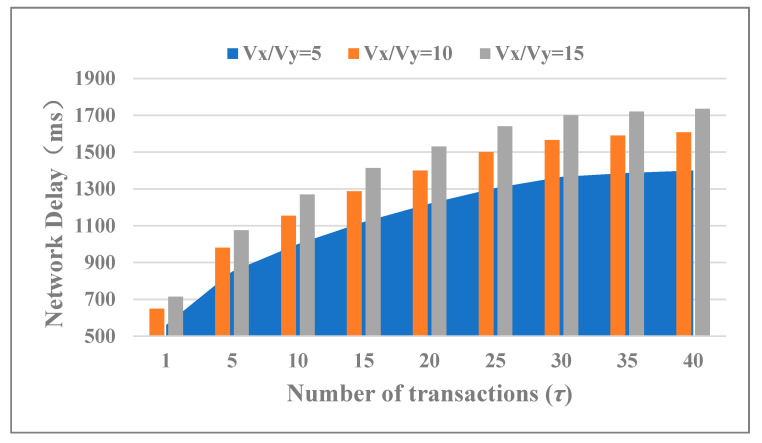
Cross-chain trading delay in different *τ* and *Vx (Vy).*

The operating efficiency and influencing factors were analyzed by the network delay caused by cross-chain smart contracts executing under different conditions. Reset the parameters of the above-mentioned: *Nx* = *Ny* = 60, *Vx* = *Vy* = 5, 10, 15 and *τ* took different values in the interval [1, 40] with a step size of 5. The actual cross-chain network delay varied with the *Vk* and *τ* as shown in Figure 5. It can be seen that the network delay increased significantly when *τ* ∈ (1, 25] and increased slowly when *τ* greater than 30. Increasing transactions in a single consensus would result in an increase in the average network delay for individual transactions although it is beneficial for improving the system throughput. Moreover, network delay increased to a certain extent with the number of nodes for cross-chain consensus verification due to the increase computational cost in consensus signature and consensus signature verification.

**Figure 6:**
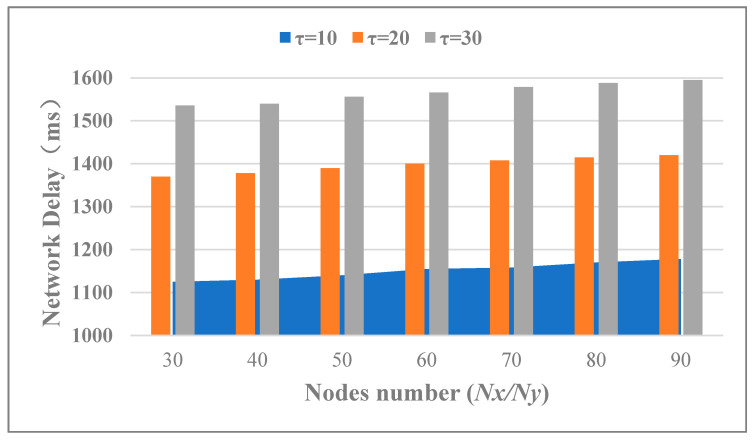
Cross-chain trading delay in different τ and *Nx (Ny).*

It is necessary to analyze the influence of the total number of nodes in a multi-microgrid system on the operation efficiency. Hence, the number of verify nodes was fixed *Vx* = *Vy* = 10, the number of transactions took *τ* = 10, 20, 30. The number of nodes *Nx* and *Ny* all took different values in the interval [30, 90] with a step size of 10. The actual cross-chain network delay varied with the N and *τ* as shown in Figure 6. It can also be seen that the number of transactions in a single consensus had a significant impact on network delay. However, the nodes number in both blockchains had no obvious effect on the network delay.

**Figure 7:**
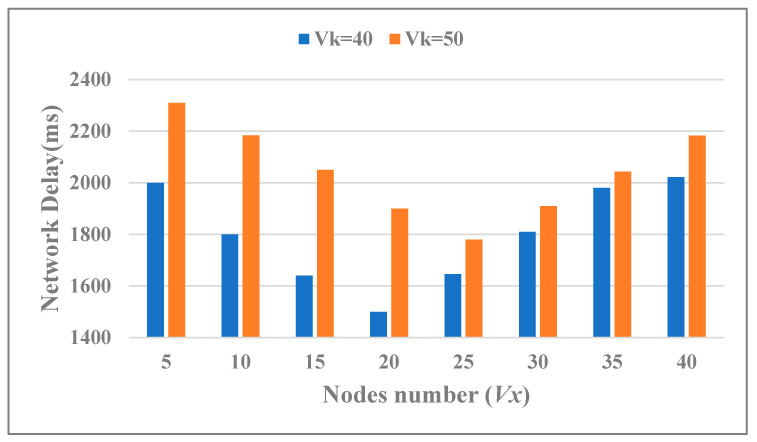
Cross-chain trading delay in different *Vx* and *Vk.*

Consider a situation of unequal rights between the partners of the blockchains in an actual application scenario. Hence, the total set of verify nodes *Vk* = 40, 50 while *Vy* took different values in the interval [5, 38] with a step size of 5. Reset the parameters of the above-mentioned: *τ* = 20, *Nx* = *Ny* = 60. The actual cross-chain network delay varied with the *Vx* and *Vy* as shown in Figure 7. It can be seen that when the total number of nodes for cross-chain consensus verification was fixed, as the number of subgroup verification nodes changes, cross-chain trading delay fluctuates regularly. Moreover, when the number of verification nodes in each subgroup was close, the network delay was lower, and vice versa. Therefore, the difference in the credibility of participating nodes in each blockchain would affect the cross-chain network delay.

According to the simulation results of the above experiments, it can be seen that the proposed cross-chain trading mode was significantly affected by two factors, including the number of transactions in a single consensus and the number of verification nodes. The number of participating nodes in each energy blockchain had little impact on the operating efficiency of the multi-microgrid system. In addition, a complete cross-chain interop communication took place within seconds in the experimental environment, which basically met the response requirements for multi-microgrid joint operation.

## 8. Conclusions

The study presents a cross-chain trading mode to fill the gap between multi-microgrid joint operation and blockchain technologies. To the best of our knowledge, this mode is the first research specifically designed for the consensus mechanism and key management in multi-microgrid cross-chain transactions.

In this paper, we proposed a novel consensus mechanism based on the dynamic evaluation of credit, which is expected to improve the efficiency and credibility of cross-chain consensus. We also designed a key management interoperability protocol to realize effective data transfer and information consensus for adaptive cross-chain transactions. This cross-chain trading mode does not need to change the underlying architecture of the original energy blockchain, and has good universality. The theoretical analysis shows that encryption/decryption, key interoperability and consensus signature are effective. Moreover, the theoretical analysis also verified that our method could offset the attack of malicious nodes, so that the system has higher security. In the case study, a series of simulation experiments proved that this trading mode takes place within seconds and meets the response requirements for energy system. Therefore, we implemented a dynamic adaptive cross-chain trading mode for multi-microgrid joint operation.

Policy support has directly affected the effective implementation of power P2P market and multilateral transactions. Cross-chain technology can promote the mutual trust between the stakeholders of each microgrid and break through the value island of each energy blockchain. This study makes full use of the blockchain distributed data structure and trust mechanism, and provides a new idea for the joint operation of multiple energy systems. The dynamic adaptive cross-chain communication can realize the mutual trust between different stakeholders and promote the wide area balance of energy. Additionally, this trading mode is likely to realize the large-scale application of blockchain technology in the energy field and promote the policy support for P2P power markets. 

However, our cross-chain mode is carried out under the condition that the scale of the energy blockchains is limited, the actual performance and the cost of building a system is unclear. In future work, we will combine the cross-chain mode with a large-scale energy system that could show its wider applicability. We also suggest that studies should be focused on the multi-energy market based on cross-chain trading technology.

## Figures and Tables

**Table 1:** Related work.

Work	Year	Technical Type	Technical Contribution	Application Scenarios
Pop et al. [31]	2019	Hash-locking	Response time and data throughput	Smart energy grid
She et al. [32]	2019	Relays method	Trust transaction	Multiple energy transaction
Ochôa et al. [33]	2020	Sidechain	Solving privacy and security problems	Smart grid
Firoozjaei et al. [34]	2020	Hybrid blockchain	Privacy-preserving and trustful	Energy transaction in IOT platforms
He et al. [35]	2020	Sidechain	Cross-system trading	Joint operation of PV and carbon markets

## References

[1] Barros E.B.C., Batista B.G., Kuehne B.T., Peixoto M.L.M. (2019). Fog Computing Model to Orchestrate the Consumption and Production of Energy in Microgrids. Sensors.

[2] Du Y., Wang Z., Liu G., Chen X., Yuan H., Wei Y., Li F. (2018). A cooperative game approach for coordinating multi-microgrid operation within distribution systems. Appl. Energy.

[3] Xia S., Chan K.W., Luo X., Bu S., Ding Z., Zhou B. (2018). Optimal sizing of energy storage system and its cost-benefit analysis for power grid planning with intermittent wind generation. Renew. Energy.

[4] Lin C., Wu W., Zhang B., Sun Y. (2017). Decentralized solution for combined heat and power dispatch through benders decomposition. IEEE Trans. Sustain. Energy.

[5] Wang J., Wang Q., Zhou N., Chi Y. (2017). A novel electricity transaction mode of microgrids based on blockchain and continuous double auction. Energies.

[6] Croce D., Giuliano F., Tinnirello I., Giarré L. (2020). Privacy-Preserving Overgrid: Secure Data Collection for the Smart Grid. Sensors.

[7] Afrakhte H., Bayat P. (2020). A self-evolving type-2 fuzzy energy management strategy for multi-microgrid systems. Comput. Electr. Eng..

[8] Xia S., Bu S., Wan C., Lu X., Chan K.W., Zhou B. (2019). A fully distributed hierarchical control framework for coordinated operation of DERs in active distribution power networks. IEEE Trans. Power Syst..

[9] Miglani A., Kumar N., Chamola V., Zeadally S. (2020). Blockchain for Internet of Energy management: Review, solutions, and challenges. Comput. Commun..

[10] Ali O., Ally M., Dwivedi Y. (2020). The state of play of blockchain technology in the financial services sector: A systematic literature review. Int. J. Inf. Manag..

[11] Noor S., Yang W., Guo M., Dama K., Wang X. (2018). Energy Demand Side Management within micro-grid networks enhanced by blockchain. Appl. Energy.

[12] Ahl A., Yarime M., Tanaka K., Sagawa D. (2019). Review of blockchain-based distributed energy: Implications for institutional development. Renew. Sustain. Energy Rev..

[13] Alladi T., Chamola V., Rodrigues J.J., Kozlov S.A. (2019). Blockchain in smart grids: A review on different use cases. Sensors.

[14] Deng L., Chen H., Zeng J., Zhang L.-J. Research on cross-chain technology based on sidechain and hash-locking. Proceedings of the International Conference on Edge Computing.

[15] Qiao R., Luo X.-Y., Zhu S.-F., Liu A.-D., Yan X.-Q., Wang Q.-X. (2020). Dynamic Autonomous Cross Consortium Chain Mechanism in e-Healthcare. IEEE J. Biomed. Health Inform..

[16] Zeng Z., Li Y., Cao Y., Zhao Y., Zhong J., Sidorov D., Zeng X. (2020). Blockchain Technology for Information Security of the Energy Internet: Fundamentals, Features, Strategy and Application. Energies.

[17] Xu X., Zeng Z., Yang S., Shao H. (2020). A Novel Blockchain Framework for Industrial IoT Edge Computing. Sensors.

[18] Wang N., Zhou X., Lu X., Guan Z., Wu L., Du X., Guizani M. (2019). When energy trading meets blockchain in electrical power system: The state of the art. Appl. Sci..

[19] Andoni M., Robu V., Flynn D., Abram S., Geach D., Jenkins D., McCallum P., Peacock A. (2019). Blockchain technology in the energy sector: A systematic review of challenges and opportunities. Renew. Sustain. Energy Rev..

[20] Reid F., Harrigan M. (2013). An analysis of anonymity in the bitcoin system. Security and Privacy in Social Networks.

[21] Wu J., Tran N.K. (2018). Application of blockchain technology in sustainable energy systems: An overview. Sustainability.

[22] Mengelkamp E., Gärttner J., Rock K., Kessler S., Orsini L., Weinhardt C. (2018). Designing microgrid energy markets: A case study: The Brooklyn Microgrid. Appl. Energy.

[23] Sikorski J., Haughton J., Kraft M. (2017). Blockchain technology in the chemical industry: Machine-to-machine electricity market. Appl. Energy.

[24] Kim S.K., Huh J.H. (2020). Blockchain of Carbon Trading for UN Sustainable Development Goals. Sustainability.

[25] Su Z., Wang Y., Xu Q., Fei M., Tian Y. (2018). A secure charging scheme for electric vehicles with smart communities in energy blockchain. IEEE Internet Things J..

[26] Schulte S., Sigwart M., Frauenthaler P., Borkowski M. Towards blockchain interoperability. Proceedings of the International Conference on Business Process Management.

[27] Scheid E.J., Hegnauer T., Rodrigues B., Stiller B. Bifröst: A Modular Blockchain Interoperability API. Proceedings of the 2019 IEEE 44th Conference on Local Computer Networks.

[28] Kannengießer N., Pfister M., Greulich M., Lins S., Sunyaev A. Bridges between islands: Cross-chain technology for distributed ledger technology. Proceedings of the 53rd Hawaii International Conference on System Sciences.

[29] Lunardi R.C., Michelin R.A., Neu C.V., Zorzo A.F. Distributed access control on iot ledger-based architecture. Proceedings of the NOMS 2018–2018 IEEE/IFIP Network Operations and Management Symposium.

[30] Deshpande A., Herlihy M. Privacy-Preserving Cross-Chain Atomic Swaps. Proceedings of the International Conference on Financial Cryptography and Data Security.

[31] Pop C., Antal M., Cioara T., Anghel I., Sera D., Salomie I., Raveduto G., Ziu D., Croce V., Bertoncini M. (2019). Blockchain-based scalable and tamper-evident solution for registering energy data. Sensors.

[32] She W., Gu Z., Yang X., Tian Z., Chen J., Liu W. (2019). Multi-energy complementary secure transaction model of heterogeneous energy blockchain. Power Grid Technol..

[33] Sestrem Ochôa I., Augusto Silva L., de Mello G., Garcia N.M., de Paz Santana J.F., Quietinho Leithardt V.R. (2020). A Cost Analysis of Implementing a Blockchain Architecture in a Smart Grid Scenario Using Sidechains. Sensors.

[34] Daghmehchi Firoozjaei M., Ghorbani A., Kim H., Song J. (2020). Hy-Bridge: A hybrid blockchain for privacy-preserving and trustful energy transactions in Internet-of-Things platforms. Sensors.

[35] He H., Luo Z., Wang Q., Chen M., He H., Gao L., Zhang H. (2020). Joint Operation Mechanism of Distributed Photovoltaic Power Generation Market and Carbon Market Based on Cross-Chain Trading Technology. IEEE Access.

[36] Singh N., Elamvazuthi I., Nallagownden P., Ramasamy G., Jangra A. (2020). Routing Based Multi-Agent System for Network Reliability in the Smart Microgrid. Sensors.

[37] Lyu Z., Yang X., Zhang Y., Zhao J. (2020). Bi-level Optimal Strategy of Islanded Multi-microgrid Systems Based on Optimal Power Flow and Consensus Algorithm. Energies.

[38] Yang H., Xiong S., Frimpong S.A., Zhang M. (2020). A Consortium Blockchain-Based Agricultural Machinery Scheduling System. Sensors.

[39] Helal S.A., Hanna M.O., Najee R.J., Shaaban M.F., Osman A.H., Hassan M.S. (2019). Energy Management System for Smart Hybrid AC/DC Microgrids in Remote Communities. Electr. Power Compon. Syst..

[40] Wan S., Li M., Liu G., Wang C. (2019). Recent advances in consensus protocols for blockchain: A survey. Wirel. Netw..

[41] Banubakode A., Patil P., Bhandare S., Wattamwar S., Muchrikar A. (2018). Key Management Interoperability Protocol-Based Library for Android Devices. Artificial Intelligence and Evolutionary Computations in Engineering Systems.

[42] Yang L.T., Huang G., Feng J., Xu L. (2017). Parallel GNFS algorithm integrated with parallel block Wiedemann algorithm for RSA security in cloud computing. Inf. Sci..

[43] Jiang Y., Shen Y., Zhu Q. (2020). A Lightweight Key Agreement Protocol Based on Chinese Remainder Theorem and ECDH for Smart Homes. Sensors.

[44] Yang B. (2017). Modern Cryptography.

